# Editorial: Molecular Dynamics and Machine Learning in Drug Discovery

**DOI:** 10.3389/fmolb.2021.673773

**Published:** 2021-04-13

**Authors:** Sergio Decherchi, Francesca Grisoni, Pratyush Tiwary, Andrea Cavalli

**Affiliations:** ^1^Fondazione Istituto Italiano di Tecnologia, Genoa, Italy; ^2^BiKi Technologies s.r.l., Genoa, Italy; ^3^Department of Biomedical Engineering, Eindhoven University of Technology, Eindhoven, Netherlands; ^4^Department of Chemistry and Biochemistry, Institute for Physical Science and Technology, University of Maryland, College Park, MD, United States; ^5^Department of Pharmacy and Biotechnology (FaBiT), Alma Mater Studiorum – University of Bologna, Bologna, Italy

**Keywords:** machine learning, molecular dynamics, computational chemistry (molecular design), drug discovery, physics-based models

The drug discovery process is very long and expensive, and many factors hamper its final success. In the attempt to accelerate a drug candidate's progress along the discovery pipeline, computational modeling represents a key tool to address the design and optimization of lead compounds. While physics-based white-box modeling (e.g., docking and molecular dynamics), has represented the standard de facto for many years in the computational chemistry community, nowadays machine learning methodologies represent a powerful modeling alternative. The deep learning paradigm in particular can be considered a black box methodology as it can be difficult to extract rules or laws from the trained model.

This Research Topic collects selected contributions that deal with both types of modeling approaches, some of which lie at the interface between the two. This “gray box” hybrid approach should not surprise as machine learning and statistical mechanics share several theoretical principles (Ferrarotti et al., 2019; Noé et al., 2019; Agliari et al., 2020; Decherchi and Cavalli, 2020; Ferraro et al., 2020; Tsai et al., 2020) as they both deal with distributions, manifolds, and hence free energies.

Molecular dynamics (MD) is based on statistical mechanics. Setting up a MD run for complex systems can be still a not trivial task, requiring continuous automation tools to allow for a wider exploitation in academic and industrial settings. In this regard, the contribution from Schneider et al. discusses the implementation of a webserver for the setup of hybrid molecular mechanics and coarse-grained simulations for Human G-Protein Coupled Receptors (GPCRs) and ligands complexes. GPCRs represent the most important class of druggable targets, hence the importance of having handy tools to setup their systematic simulations. Analyzing and understanding MD outcomes can then be rather complicated, mainly because of the large amount of raw data. Bunker and Rog present a review on the mechanistic understanding of MD generated data for drug delivery in pharmaceutical research. Mechanistic interpretations can be supported by proper machine learning tools: it is often convenient to devise/use clustering, projections or feature extraction algorithms to extract actionable knowledge. This greatly facilitates the interpretation of results and can also allow to define order parameters in some cases, often dubbed collective variables in the MD realm. In the contribution from Arthur et al. Authors devise a combination of MD simulations of proteins and hierarchical pharmacophore features extraction. This strategy represents a smart and widely applicable paradigm (Spyrakis et al., 2015) which combines MD sampling (to recover some of the target flexibility) with a non-dynamical tool (e.g., virtual screening, static docking etc.). While this paper derives features directly for drug discovery, Spiwok and Kriz propose a more general approach. The Authors present a new machine learning algorithm, named time lagged t-SNE, which is able to explicitly take into consideration different time scales in the simulation. Such detection (and acceleration) of slow and fast time scales is very important in drug discovery; in protein ligand binding, for instance, several phenomena of interest (e.g., unbinding) happen at very large time scales as they are rare events.

At variance of qualitative analysis for devising mechanistic hypothesis, quantitatively converged estimations are the only possible path to try predicting physical observables. Statistical mechanics research has developed powerful theories for quantifying observables of interest and, in the drug discovery realm, binding free energy is a key physicochemical quantity. Despite the great improvements in the last 20 years both in theoretical and technological terms, predictive free energy computations still remain partially elusive for many reasons, such as the massive computing power needed for convergence, the force field accuracy, possible numerical instabilities in some cases, and the partial disconnection between experimental observables and what is effectively estimated by computations. Hall et al. discuss this important aspect, namely the relationship between kinetics estimated via the weighted ensemble method and the experimental affinity. While the general relationship between experimental kinetics and affinity is known, when it comes to simulations the situation becomes subtler. Authors show that some correction terms (for instance finite-size effects) whose energetic contribution is not negligible arise. These corrections allow to get much more accurate free energy estimations derived from kinetics rates estimated via the weighted ensemble. Free energy (or kinetics) simulations can be quite expensive, hence approximate methods can be devised. Fully data driven or approximate physics-based models have proved more or less effective in granting a compromise between accuracy and efficiency. The Linear Interaction Energy method is one of such approximate physics-based strategy and Rifai et al. discuss recent advances of this methodology. Interestingly, from a machine learning perspective, this kind of methodologies could be ascribed to the previously mentioned “gray box” approaches. They start from a physically sound *ansatz* and then switch to a data-driven style to tune the remaining parameters to save computing time. End-to-end data-driven attempts are also possible, often based on *ad-hoc* engineered features to describe the ligand and the protein, on which machine learning can be applied. This is what happens in the contributions of Holderbach et al. and in Parks et al. where physicochemical features are first devised and then used to predict affinity.

This collection of articles has dealt with many, and often interconnected, algorithmic approaches to speed-up the discovery of new drugs and the estimation of key observables such as free energy. We believe this collection will be useful to computational and medicinal chemists willing to apply recent *in-silico* methodologies, ranging from pure MD to fully data-driven approaches. We thank all Authors, co-Authors, and Reviewers for their contribution to this Research Topic and acknowledge Frontiers Team members'support.

## Author Contributions

SD conceived the topic, managed the review process, and wrote the editorial. FG, PT, and AC contributed to the review process and to the editorial. All authors contributed to the article and approved the submitted version.

## Conflict of Interest

SD and AC are co-founders of BiKi Technologies s.r.l. a company which commercializes computational chemistry tools for drug discovery. The remaining authors declare that the research was conducted in the absence of any commercial or financial relationships that could be construed as a potential conflict of interest.

## References

[B1] Agliari E. Barra A. Sollich P. Zdeborová L. (2020). Machine learning and statistical physics: preface. J. Phys. A Math. Theoretic. 53:500401. 10.1088/1751-8121/abca75

[B2] Decherchi S. Cavalli A. (2020). Thermodynamics and kinetics of drug-target binding by molecular simulation. Chem. Rev. 120, 12788–12833. 10.1021/acs.chemrev.0c00534 33006893 PMC8011912

[B3] Ferraro M. Decherchi S. De Simone A. Recanatini M. Cavalli A. Bottegoni G. (2020). Multi-target dopamine D3 receptor modulators: actionable knowledge for drug design from molecular dynamics and machine learning. Eur. J. Med. Chem. 188:111975. 10.1016/j.ejmech.2019.111975 31940507

[B4] Ferrarotti M. J. Rocchia W. Decherchi S. (2019). Finding principal paths in data space. IEEE Transac. Neural Netw. Learn. Syst. 30, 2449–2462. 10.1109/TNNLS.2018.2884792 30596587

[B5] Noé F. Olsson S. Köhler J. Wu H. (2019). Boltzmann generators: sampling equilibrium states of many-body systems with deep learning. Science 365:eaaw1147. 10.1126/science.aaw1147 31488660

[B6] Spyrakis F. Benedetti P. Decherchi S. Rocchia W. Cavalli A. Alcaro S.. (2015). A pipeline to enhance ligand virtual screening: integrating molecular dynamics and fingerprints for ligand and proteins. J. Chem. Inf. Model. 55, 2256–2274. 10.1021/acs.jcim.5b00169 26355717

[B7] Tsai S.-T. Kuo E.-J. Tiwary P. (2020). Learning molecular dynamics with simple language model built upon long short-term memory neural network. Nat. Commun. 11:5115. 10.1038/s41467-020-18959-8 33037228 PMC7547727

